# *In silico* analyses and global transcriptional profiling reveal novel putative targets for Pea3 transcription factor related to its function in neurons

**DOI:** 10.1371/journal.pone.0170585

**Published:** 2017-02-03

**Authors:** Başak Kandemir, Ugur Dag, Burcu Bakir Gungor, İlknur Melis Durasi, Burcu Erdogan, Eray Sahin, Ugur Sezerman, Isil Aksan Kurnaz

**Affiliations:** 1 Gebze Technical University, Department of Molecular Biology and Genetics, Kocaeli, Turkey; 2 Yeditepe University, Biotechnology Graduate Program, Kayisdagi, Istanbul, Turkey; 3 Sabanci University, Faculty of Engineering and Natural Sciences, Istanbul, Turkey; 4 Gebze Technical University, Institute of Biotechnology, Kocaeli, Turkey; Saint Louis University School of Medicine, UNITED STATES

## Abstract

Pea3 transcription factor belongs to the PEA3 subfamily within the ETS domain transcription factor superfamily, and has been largely studied in relation to its role in breast cancer metastasis. Nonetheless, Pea3 plays a role not only in breast tumor, but also in other tissues with branching morphogenesis, including kidneys, blood vasculature, bronchi and the developing nervous system. Identification of Pea3 target promoters in these systems are important for a thorough understanding of how Pea3 functions. Present study particularly focuses on the identification of novel neuronal targets of Pea3 in a combinatorial approach, through curation, computational analysis and microarray studies in a neuronal model system, SH-SY5Y neuroblastoma cells. We not only show that quite a number of genes in cancer, immune system and cell cycle pathways, among many others, are either up- or down-regulated by Pea3, but also identify novel targets including ephrins and ephrin receptors, semaphorins, cell adhesion molecules, as well as metalloproteases such as kallikreins, to be among potential target promoters in neuronal systems. Our overall results indicate that rather than early stages of neurite extension and axonal guidance, Pea3 is more involved in target identification and synaptic maturation.

## Introduction

ETS domain transcription factors are characterized by an evolutionarily-conserved ETS domain of about 85 amino acids that facilitates binding to DNA sequences with a central GGAA/T core consensus and flanking nucleotides [1]. Around 30 members of the ETS proteins have been identified in mammals and are categorized within several subfamilies. Among them, PEA3 subfamily members, most notably Pea3/ETV4, Erm/ETV5 and Er81/ETV1, also bind to the DNA core sequence GGAA/T [2], and contain an acidic activation domain in the N-terminus as well as a C-terminal activation domain [3]. Pea3 family members are involved in several processes, including breast cancer, prostate cancer [4], motor neuron connectivity and dendritic arborization [5] as well as neuronal differentiation [6,7].

Pea3/ETV4 is highly expressed in Her/Neu expressing breast cancer cells and tissues, and the major targets for Pea3/ETV4 previously identified in these tissues were matrix metalloprotease enzymes, particularly MMP1, MMP2 and MMP9, which are required for the initiation of cell migration [8]. In addition, overexpression of Pea3/ETV4 was shown to result in increased levels of vimentin [9], the intercellular adhesion molecule ICAM-1 [10,11], osteopontin [12], vascular endothelial growth factor and cyclooxygenase-2 [13], thus providing evidence for the importance of PEA3/ETV4 in tumor formation and metastasis. But although much is known about how PEA3/ETV4 is involved in breast or prostate cancer [14], very little is understood about how it regulates motor neuron connectivity, retinal development or ganglion cell differentiation [15,16], or indeed which promoters are Pea3 targets in the nervous system. In *C*. *elegans*, ETS protein Ast-1 (axon steering defect-1) was shown to regulate dopaminergic neuron differentiation through regulating some of the major dopaminergic genes with *ets* motifs [17], but no such targets are yet identified for ETS proteins in mammalian dopaminergic differentiation. On the other hand, cadherin-8, ephrin receptor 4 (Ephr4) and semaphorin-3E were shown to be Pea3 targets in neurons ([16, 18]; also confirmed in this study).

To reveal the possible neuronal targets of Pea3, in this study we have taken the following complementary approach:

Firstly, we have manually curated neural differentiation- and axon guidance-related promoter sequences and analyzed the selected promoter reqions for the selected transcription factor.

Secondly, we have developed an automated tool to identify all promoters that contain the binding site for a given transcription factor. Although this approach is less labor-intensive compared to the prior strategy of manual curation, it is limited to the entries within the existing promoter databases. Yet, our study shows that there is significant overlap between these two *in silico* target identification approaches.

Thirdly, we have conducted microarray analyses, where we have not only confirmed a subset of genes identified in the above-mentioned *in silico* analyses, but also identified many more potential novel targets for Pea3 transcription factor. These novel targets include several genes that function in cytoskeletal organization, axon guidance, cell migration, ion channels, enzymes and signaling pathway components, as well as many others. KEGG pathway-based analysis of microarray data also showed a significant number of novel genes in neurotrophin signaling pathway, MAPK pathway, glioma pathway and long-term potentiation, among many others. A small subset of these were further analyzed and confirmed through qRT-PCR analysis, and *in silico* tools predicted high affinity binding sites for Pea3 in their promoters.

One important finding is the mixed nature of Pea3 transcriptional activity—while it activated some of these novel target promoters, it was found to repress others. We do not as yet know the detailed mechanism of this regulation, ie whether there are coactivators or corepressors involved, or if posttranslational modifications of Pea3 render it as an activator or a repressor, or indeed whether there is an indirect regulation through activation of miRNA genes that in turn repress some of these promoters [21]. Nonetheless, the analysis of the small subset or target genes presented in this study indicate that rather than regulating axonal outgrowth and guidance, Pea3 is more likely to be involved in target recognition, growth cone collapse, and/or synaptic maturation, and involved in endocytosis as well as synaptic vesicle cycle. This is in line with previous findings that Pea3 family members function at later rather than earlier stages of neuronal differentiation.

## Materials and methods

### Curation of potential target promoters for analysis

**Fig 1:**
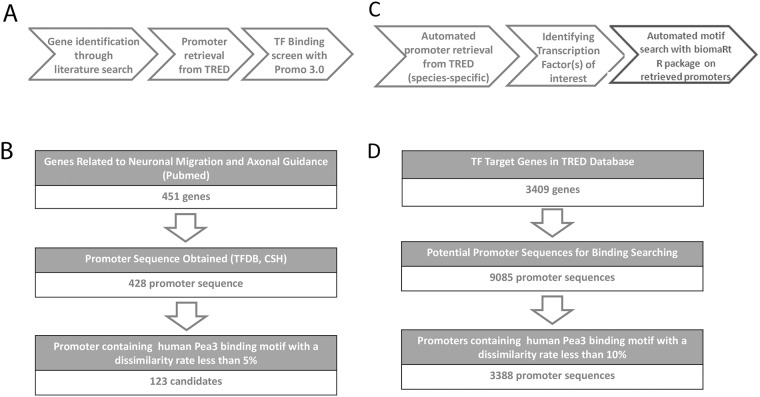
(a) and (b) Experimental flowchart and summary of manual curation-based promoter analysis; (c) and (d) Experimental flowchart and summary of automated promoter analysis. (a) Genes of interest were manually curated and determined using PubMed and NCBI Gene tools; corresponding promoters were retrieved from TRED database, followed by screening for transcription factor (TF, in this case Pea3) binding using Promo 3.0 tool (see text for details); (b) With respect to neuronal migration and axonal guidance, a total of 451 genes were identified, for which only 428 promoters were retrieved. Upon analysis, only 123 possible candidate promoters were identified to contain Pea3 binding motif with a dissimilarity rate of less than 5%; (c) upon development of the automation program, it was used to retrieve promoters from TRED in a species-specific manner, followed by identification of the transcription factor(s) of interest by the user, whose binding motifs were searched using Promo 3.0 tool (see text for details); (d) a total of 3409 genes and a corresponding 9085 promoters (multiple promoter entries were possible for some genes) were retrieved and analyzed, which yielded 3388 promoter sequences that contain Pea3 binding motif with a dissimilarity rate of less than 10%.

Since this study is concerned primarily with identification of novel target promoters of Pea3/ETV4 with respect to the nervous system development, we were mainly focused on potential target genes involved in “neuronal migration” and “axonal guidance”; these two phrases were used as our gene search parameter. The genes searched for these criteria have been identified by means of “Gene” tool of NCBI (http://www.ncbi.nlm.nih.gov/gene/). The promoter sequences that correspond to these curated set of genes were then retrieved from the Transcriptional Regulatory Element Database, TRED (http://rulai.cshl.edu/cgi-bin/TRED/tred.cgi?process=home; [19]). This website has a genome-wide database for the promoter sequences, and using the transcription start site (TSS) setting, the target promoter sequences were displayed from -700 to +300 base pairs relative to TSS (Fig 1a).

### Analysis of the promoter sequences for Transcription Factor Binding

The promoter sequences manually obtained from TRED were analyzed with PROMO 3.0 (http://alggen.lsi.upc.es/cgi-bin/promo_v3/promo/promoinit.cgi?dirDB=TF_8.3; Fig 1a). PROMO 3.0 tool analyzes the promoter regions for binding by a selected transcription factor, and displays the results with a “dissimilarity rate” [20]. Dissimilarity rate simply implies the variance between the binding motif of the transcription factor and the nucleotide sequence on the promoter as percentage by regarding the binding matrices. From this point of view, the smaller dissimilarity rates are the indicators of higher possibility for Pea3/ETV4 binding (0% dissimilarity rate shows 100% identity to consensus motif). To confirm the reliability of this method, promoter sequences for matrix metalloproteases MMP3 and MMP9 as well as Vascular Endtothelial Growth Factor (VEGF), the known targets for Pea3/ETV4 [13, 22, 23] were used as positive controls, with dissimilarity rates determined to be 0% as expected (data not shown).

### Development of a promoter analysis tool

While the above manual analysis requires the user to find and define selected subset of promoter sequences from any nucleotide database and analyze it for presence or absence of one particular Transcription Factor (TF) binding motif (promoter by promoter), an automated tool was designed to obtain the promoter sequences of all human genes (user-defined range, eg 1000 bp upstream) using biomaRt R package [24,25] http://www.ensembl.org/info/data/biomart/biomart_r_package.html).

In the first step, the automation tool retrieves all human protein coding genes with their Entrez IDs and gene names from the Ensembl database (http://www.ensembl.org). In the second step, using the human gene list, promoter regions are selected among these sequences according to the user defined criteria. In the third step, using MotifDB R library [26] (http://bioconductor.org/packages/release/bioc/html/MotifDb.html), position weight matrices (PWM) for any transcription factor are retrieved [27]. (For our specific application in this study, etv4 PWM is retrieved to define Pea3 binding motifs on promoters.) The algorithm then searches in the promoter regions for the presence of subsequences with a minimum matching score of 80% to the PWM selected. All promoters with predicted etv4 binding motifs are reported in this study.

### Cell culture and transfection

SH-SY5Y human neuroblastoma cell line (ATCC(^®^) CRL-2266^™^) is typically maintained in the high glucose DMEM (Gibco, 1129855) supplemented with 10% Fetal Bovine serum (Life Technologies, 10500–064) in the presence of penicillin, streptomycin, L-Glutamine and amphotericin B (Biological Industries, 03-033-1B) and primocin (Invivogen, ant-pm-1). For transfection, SH-SY5Y cells were seeded at 1.5 million cells per 10 cm diameter dish, and 24 hr later transfected with either pCDNA3 and pCDNA3-mPea3-VP16 (courtesy of Prof. A.D. Sharrocks) using the PEI reagent (CellnTech), in 3 replicas per sample.

### RNA isolation, cDNA synthesis, Reverse Transcription Polymerase Chain Reaction (RT-PCR) and Real-Time PCR

Total cytoplasmic RNA is commonly prepared using RNAeasy kit (Qiagen, cat no 74104) as per manufacturer's instructions. 1 μg RNA was used for each first strand cDNA synthesis reaction (M-Mu-LV-Rtase, Roche) as per manufacturer’s instructions, using random primers (Boehringer Mannheim). The amount of cDNA used was standardized using GAPDH and linear range was determined. Typically the RT-PCR reactions were performed using 10–50 ng cDNA template in 20 μl reaction with BioTaq polymerase at 54.5°C for 30 cycles. For conventional PCR, the products were resolved in 2.5% Nu-Sieve) agarose gels and were analyzed using QuantityOne imaging software (BioRad).

On the other hand, 40 ng cDNA template in 10 μl reaction with IQ SYBR green super mix (BioRad, cat no 170–8880) was used for Real-time polymerase chain reaction (qRT-PCR) and carried out using a CFX96 Touch Real-Time PCR detection system. To evaluate whether the difference in gene expression level between control and transfected cells was significant, the efficiency (E) -corrected delta cycle threshold (ΔCt) method was used according to the formula:

$${\text{relative quantity (RQ)}}_{\text{target}}=\frac{Etarget^{Ct\left(pCDNA3\right)-Ct\left(Pea3-VP16\right)}}{Egapdh^{Ct\left(pCDNA3\right)-Ct\left(Pea3-VP16\right)}}$$

The RQ values thus calculated were then transformed on a log2 scale to achieve normal distribution of the data and the resulting distributions were tested against the null-hypothesis of equal mRNA level in control and transfected cells (i.e., a population mean of 0.0) using two-tailed one-sample Student’s t-tests. An α-level of ≤ 0.05 was applied for all comparisons to determine statistical significance.

The list of primers used in RT-PCR and qRT-PCR are shown in Table 1.

**Table 1:** The list of primers used in qRT-PCR analyses (* primer sequences obtained from Pratt and Kinch, 2003).

Gene	Forward Primer (5’>3’)	Reverse Primer (5’>3’)
KLK2	GATTGTGGGAGGCTGGGAGTGTGAG	GGACAGGAGATGGAGGCTCACACAC
KLK3	AGC GTG ATC TTG CTG GGT CG	CCTTGAAGCACACCATTACAGAC
KLK4	ATT GTT CTG CTC GGG CGT CCT G	GGGTCTGTTGTACTCTGGGTGC
KLK5	GCA TCC ACA GTG GCT GCT CA	TGAGCATGAGGTTGTTAGAGTGGC
KLK6	GGG TCC TTA TCC ATC CAC TGT G	TGGCGGCATCATAGTCAGGGTG
KLK7	GGA ACC ACC TGT ACT GTC TCC	TTTCTTGGAGTCGGGGATGCC
KLK8	TTG TAG GTG GCA ACT GGG TCC	CTGGTCACGCAGTTGAAGAAGC
KLK9	CTC AAC CTC AGC CAG ACC TGT GT	TGCTGTCCGAGATGTGTCCAG
GRIK3	TGAACCTCTACCCCGACTACG	ATGGGGAGCTGACGGATCTTCAG
GLUD2	GAATGCTGGAGGAGTGACAGTATC	GCAGAACGCTCCATTGTGTATG
EFNB2	GCAAGTTCTGCTGGATCAAC	AGGATGTTGTTCCCCGAATG
EFNB1	GGAGGCAGACAAACATGTCA	GAACAATGCCACCTTG
EFNA3	CCACTCTCCCCCAGTTCACCATG	GCTAGGAGGCCAAGAACGTC
EPHA1	CTGCTGCTTGGTGCAGCCTTG	GCTTCAGCCACAGCTTGTCCTCTCG
EPHA2*	ATGGAGCTCCAGGCAGCCCGC	GCCATACGGGTGTGTGAGCCAGC
L1CAM	GCTGGTTCATCGGCTTTGTG	GTCTCATCTTTCATCGGTCGG
PTK2B	GATGACCTGGTGTACCTCAATG	GTGTGAAGCCGTCAGCATCTG
UNC5A	GCCTTCAAGATCCCCTTCCTC	CTGGGCTTGGAGGCAAAGAAG
SEMA4C	CTGAGAGGACCTTGGTGTACC	GGTGAAGCCGAGTTGGAGAAG
NGFR	GAGAAAAACTCCACAGCGACAGTG	GGTAAAGGAGTCTATGTGCTCGG
FGFR1	GTACATGATGATGCGGGACTGCTG	GAGAAGACGGAATCCTCCCCTGAG

### Microarray and data analysis

For microarray analysis, SH-SY5Y cells were transfected as described above, and 48 hr after transfection RNA samples were isolated using Ambion Tri-pure RNA isolation kit, checked for quality, converted to cDNA and confirmed for Pea3 expression as described above. Thereafter, RNA was converted to cDNA using the *Superscript Double-stranded cDNA Synthesis (Invitrogen)* Kit and labeled with *NimbleGen One Color DNA Labeling* (NimbleGen, Roche). The labeled cDNA were hybridized to NimbleGen Human Gene Expression Array 12x135K (NimbleGen, Roche), which covers 45.033 genes with 3 probes per gene, containing 12 arrays per slide. After hybridization, slides were scanned using Genepix 4000B scanner and analyzed with NimbleScan 2.5 software using three arrays from pCDNA3-transfected cell as reference samples. The averaged fold changes and p values for each gene were calculated, and genes which were up- or down-regulated, with FDR (False Discovery Rate) adjusted p value of 0.05 or less were assumed to be significant [28]. Data was submitted to EBI ArrayExpress, accession E-MTAB-5324.

Gene IDs were converted to official gene symbol, then *Kyoto Encyclopedia of Genes and Genomes* (KEGG) pathway tools were used for functional enrichment of the list of genes and identification of affected pathways and processes. KEGG pathway tools were analyzed through both PANOGA online tool (http://panoga.sabanciuniv.edu/index.html; Sezerman Lab) using STRING protein protein interaction database (http://string-db.org/newstring_cgi/show_input_page.pl; free). Genes with p-values (significance values) smaller than 0.05 were listed and used for further analysis. PANOGA maps the list of genes and their significance values to STRING PPI network and identifies active subnetworks involving most of the affected genes by PEA. Then it identifies affected KEGG pathways within these subnetworks and assigns significance to them based on hypergeometric distribution.

### Weblogo analysis

Putative Pea3 binding motifs on a specific subset of promoters were further analyzed using Weblogo version 2.8.2 (http://weblogo.berkeley.edu/logo.cgi). This freely available online tool generates a graphical representation of amino acids or nucleic acids after multiple sequence alignment, where the overall height of the particular residue indicates the degree of conservation of that residue in all sequences analyzed.

### Chromatin immunoprecipitation (ChIP) assay

SH-SY5Y cells were plated in 150 mm diameter dishes and twenty four hours later transfected with either empty pCDNA3 or Pea3-VP16 expression plasmid, as described above. Forty eight hours after transfection cells were cross-linked with 1% formaldehyde and lysed in lysis buffer (85 mM KCl, 0,5% NP-40, 20 mM Tris-HCl pH8.0, protease inhibitor cocktail). The lysates were sonicated using Bioruptor Pico (Diagenode) in nuclei isolation buffer (100 mM HEPES, 1,5 mM MgCl_2_, 10 mM KCl, 1 mM DTT, protease inhibitor coctail). 10% v/v of the sheared DNA was separated as input, and rest of the sample was precipitated using 30 μl of anti-Flag M2 affinity resin (Sigma) or normal mouse IgG (Santa Cruz, sc-2025) overnight. Immunoprecipitated chromatin was washed and eluted in elution buffer (20%SDS, 1M NaHCO_3_). Cross-linking of proteins and DNA was reversed and treated with RNaseA and proteinase K. DNA was then purified using MEGAquick-spin^™^ Total Fragment DNA Purification Kit (Intron). Enrichment at promoter sites was detected by qPCR using iTaq Universal SYBR Green Supermix (BioRad). MMP9 promoter region was used as a positive control, and FGFR1 intron region harboring no *ets* motifs served as negative control (data not shown). Primers used in ChIP qPCR are listed in Table 2. ChIP-qPCR data was analyzed according to the formula

$$\text{Relative ChIP binding}={\text{2}}^{-\left(Ct[IP]-Ct[Input\times DF]\right)}\times 100\%$$

where Ct is the cycle threshold, IP is the qPCR intensity units obtained from qPCR of chromatin IP samples, Input is that obtained from input, and DF is the dilution factor.

**Table 2:** The list of primers used in ChIP qPCR analyses.

Gene ID	Forward (5'-3')	Reverse (5'-3')
Akt1—1	CAGGAAGGCCCATCTGGAAG	CCCTCACCTGAGCACACTTT
Akt1—2	CCCAGGAGGTTTTTGGGCTT	CGTTTGCTCTCCCTGTCCAT
EPHA1—1	CCAACCAGATCAGCCCATGT	CGAGTGGAAGTGCAGGATGT
EPHA1—2	GAGTGGCTCGAGTCCATACG	CTGTGGGCAAGGAAGGGTG
EPHA1—3	AAGGTCGCTCATGGTCACTC	TAACCCCTCAGCTCCCTCC
EPHA2—1	GGGTACCTCAAGCCCCATTT	CAAGCATCTTGCAAAGGCCC
EPHA2—2	AACATTCGTGAGCTGGGGAC	AGACTGAAAGCCAAGATCGGT
FGFR1	TCTCGCAACAGGAAGGAACC	GGGGTTGTGAGTGGAGACAG
L1CAM	GGAGCTCCATACACACGCTG	TCAGACGATAGGGAGGGCAG
MMP2	CCCCTGTTCAAGATGGAGTC	CCCAGGTTGCTTCCTTACCT
Negative	GGACGTGGAGGGCTAGGTTA	TTAACGACCGTGGGTTGTCC
SEMA4C—1	GCCCAAGTGCACCTACGTC	TCCAAAGTGAAGGTGAGCATGT
SEMA4C—2	GTCCCTATGACCCAGCTAAGG	ACCATCTATGGGAGACAGAGGT

## Results and discussion

The aim of this combinatorial study was to identify novel transcriptional targets for Pea3 with respect to its neuron-specific functions. To that end, our first approach was an *in silico* analysis through manual curation of predicted target promoters for Pea3/ETV4 (Fig 1a). 404 human genes related to neuronal migration and 47 human genes related to axonal guidance were manually curated, and promoter sequences for 428 of these were found through nucleotide databases (Fig 1). Out of these, 123 candidate promoters crossed the threshold (5% dissimilarity rate) for Pea3/ETV4 binding (Fig 1b).

When the promoters that contain lower than 5% dissimilarity score for either mouse or human Pea3 binding motifs for both neuronal migration and axonal guidance were compared, it was seen that 19 promoters were common in both functions (Table 3). Among these, 6 of them were seen to be related to adhesion, 10 related to cell-to-cell signaling, 2 were considered to be structural, and 1 was a transcription factor (Table 3). The dissimilarity scores of the promoters of these genes (either from human or mouse promoter database) for Pea3 binding are listed in Table 3, and may differ in a species-specific manner; for example, for SLIT2, Slit homolog 2, mouse Pea3 binding dissimilarity rate was found to be 3,94%, whereas that for human Pea3 was as low as 0,43% (Table 3). SLIT2 is an axonal guidance molecule that appears to be essential for midline crossing in the midbrain as well as spinal cord by modulating the cell’s responses to Netrin (http://www.genecards.org/cgi-bin/carddisp.pl?gene=SLIT2), but also important in kidney, inflammation, angiogenesis and glioma migration [29–32], all of which are processes where Pea3/ETV4 is implicated. On the other hand for KAL1, Kallman syndrome 1, the scores were just the opposite, 0,63% for mouse Pea3 and 9,45% for human Pea3 binding (Table 3). KAL1 gene codes for the axonal guidance protein called anosmin 1 particularly involved in the developing brain, and is known to be involved in neurite branching [33] (http://www.genecards.org/cgi-bin/carddisp.pl?gene=ANOS1&keywords=KAL1).

**Table 3:** The putative Pea3 target genes identified through manual curation with respect to neuronal migration and axon guidance.

Gene symbol	Gene name	Accession #	mPea3	hPea3	Function of genes	REFS
BDNF	Brain Derived Neurotrophic factor	8188	0,63	N/A	Growth factor activity (cell-cell signaling)	[42,85]
CDK5R1	Cyclin Dependent Kinase 5 regulatory subunit 1	115721	N/A	9,45	Calcium ion binding, protein kinase activity (cell-cell signaling)	[87]
CNTN2	Contactin 2	1782	3,94	9,24	Carbonhydrate and glycoprotein binding (adhesion)	[88]
EphA8	Ephrin Receptor A8	318	3,94	N/A	ATP binding, nucleotide binding and receptor activity (cell-cell signaling)	[43,89]
EphB2	Ephrin Receptor B2	323	N/A	9,67	Ephrin receptor activity, nucleotide binding, protein tyrosine kinase activity (cell-cell signaling)	[90]
GNAI2	Guanine nucleotide binding protein (G protein) alpha inhibiting activity polypeptide 2	29399	1,70	9,67	GTPase activity, signal transducer (cell-cell signaling)	[91]
KAL1	Kallmann syndrome 1 sequence	44617	0,63	9,45	Extracellular matrix structural constituent (structural)	[34, 92]
L1CAM	L1 Cell adhesion molecule	113184	0,63	0	Identical protein binding (adhesion)	[34,93]
MAPK8IP3	mitogen-activated protein kinase 8 interacting protein 3	14609	3,94	7,14	MAP kinase scaffold activity (cell-cell signaling)	[94]
MYH10	myosin, heavy chain 10, non-muscle	19064	6,61	N/A	Actin binding, microfilament motor activity (structural)	[95]
NCAM1	Neural Cell Adhesion Molecule 1	7078	0	0,43	Identical protein binding (adhesion)	[96]
NEUROG2	Neurogenin 2	32273	0,63	0,21	Sequence-specific DNA binding (Transcription Factor)	[97]
NGFR	Nerve growth factor receptor	17440	1,70	9,24	Receptor and signal transducer activity (cell-cell signaling)	[98]
NRCAM	Neuronal cell adhesion molecule	38906	8,32	9,67	Ankyrin binding (adhesion)	[99]
Nrp1	Neuropilin 1	5859	3,31	9,24	Growth factor binding (cell-cell signaling)	[100]
NTF3	Neurotrophin 3	8613	0	7,14	Receptor binding (cell-cell signaling)	[101]
PTK2	Protein Tyrosine Kinase 2	116986	0,63	0	Nucleotide binding and signal transducer activity (cell-cell signaling)	[102]
SEMA4A	Semaphorin 4A	1354	3,94	9,67	Receptor activity (adhesion)	[86, 103]
SLIT2	Slit Homolog 2	116382	3,94	0,43	GTPase inhibition, Roundabout binding, calcium ion binding (adhesion)	[29,51,104]

The promoters that consistently had lowest dissimilarity rates for both mouse and human Pea3/ETV4 binding were considered as more likely targets for a consistent and conserved Pea3-dependent regulation: Protein tyrosine kinase 2 (PTK2) exhibited dissimilarity scores of 0,63 for mouse and 0 for human Pea3/ETV4 binding; L1 cell adhesion molecule (L1CAM) exhibited 0,63 dissimilarity score for mouse Pea3 and 0 for human ETV4 binding; Neural cell adhesion molecule 1 (NCAM1) showed 0 dissimilarity for mouse and 0,43 for human Pea3/ETV4; and Neurogenin 2 (NEUROG2), 0,63 for mouse and 0,21 for human Pea3/ ETV4 binding. Among these, L1CAM was particularly interesting since it was shown to be present in a complex with KAL1-FGFR in regulating neurite branching [33], and also known to regulate axon-axon interaction [34]–KAL1 being the other putative target identified through this method (discussed in the previous paragraph).

Some promoters were only analyzed for mouse or human Pea3 binding, including ephrin receptor B2, ephrin receptor A8, CDK5 regulatory subunit 1, BDNF, and myosin heavy chain 10, since promoter sequence from only one organism’s genome could be accessed (Table 3). Ephrins and their receptors are also interesting targets for Pea3 regulation, since they are not only involved in cell guidance and migration during axonal development, but also in glioblastoma progression [35, 36].

### Automated promoter analysis tool

The above analysis was based on a manually curated set of promoters that were identified with respect to their involvement in neuritogenesis, migration and axonal guidance. We next wanted to address whether the automated analysis tool that we developed that screens for an entire promoter database for putative Pea3 binding in an unbiased fashion would result in a similar set of potential target promoters. When this promoter analysis tool was employed (see Materials and Methods for details; Fig 1c), a total of 9085 promoter sequence entries for 3409 genes were retrieved and analyzed for putative Pea3/ETV4 binding (Fig 1d). For this particular genome-wide *in silico* analysis, a higher dissimilarity score of 10% was set as threshold, which resulted in the identification of 3388 promoter sequences positive for Pea/ETV4 binding motifs (Fig 1d).

When the results from this automated tool was compared with manually identified targets for Pea3/ETV4, 57 genes were found to be overlapping, 15 of which had lower than 5% dissimilarity for Pea3/ETV4 binding in both mouse and human promoters (Table 4). Out of these, ANGPT-1 (angiopoietin) is widely known as an endothelial growth factor, and yet it was shown to protect neurons from apoptosis [37]. Similarly, CX3CR1 (chemokine C-X3-C motif receptor 1) is implicated in neuronal survival, where knockout of CX3CR1 in microglia was shown to prevent neuronal loss [38]. Integrin-like kinase (ILK) mediates survival and synaptic plasticity of hippocampal neurons [39]. And the tumor suppressor protein TP53 was shown to play a role in the survival of neural progenitor cells [40].

**Table 4:** The putative Pea3 target genes identified *in silico* that are overlapping in both automated analysis and manual curation.

Gene symbol	Gene name	Accession #	mPea3	hPea3
ACTN2	Actinin, alpha 2	2058	0	9,45
ADAM10	ADAM metallopeptidase domain 10	14166	0	9,45
ANGPT-1	Angiopoietin	113693	0	0,43
APC	Adenomatous polyposis coli	33308	0,63	9,45
BMP2	Bone morphogenic protein 2	113729	8,32	9,67
CDH1	Cadherin 1, type 1, E-cadherin (epithelial)	15369	3,94	N/A
CDK5R1	Cyclin-dependent kinase 5, regulatory subunit 1	115721	N/A	9,45
CIB1	Calcium and integrin binding 1 (calmyrin)	13845	4,38	0,21
CSF1	Colony stimulating factor 1 (macrophage)	112668	1,07	9,45
CST3	Tachykinin receptor 1	25458	7,25	N/A
CX3CR1	Chemokine (C-X3-C motif) receptor 1	30958	0,63	0
DCLK1	Doublecortin-like kinase	11447	3,94	7,14
DIAPH1	Diaphanous homolog I (Drosophila)	34180	1,7	0,21
DPYSL2	dihydropyrimidinase-like 2	39825	0,63	7,14
DRD5	Dopamine receptor D5	31284	1,07	7,14
DYX1C1	Dyslexia susceptibility 1 candidate 1	124118	8,32	9,45
EGR1	Early growth response 1	33479	3,31	7,14
FES	Feline sarcoma oncogene	13679	3,31	9,45
GMIP	GEM interacting protein	120115	3,94	N/A
GNB1	Guanine nucleotide binding protein	4136	0	9,24
GNB2L1	G protein, beta polypeptide 2-like 1	33870	N/A	6,93
HSPA4	Heat shock protein 70 kDa protein 4	33414	3,94	0,21
HSPB1	Heat shock 27 kDa protein 1	37879	1,7	N/A
IGFBP3	Insulin-like growth factor binding protein 3	39292	3,31	9,45
ILK	Integrin-linked kinase	6243	0,63	0,43
INS	Insulin	8437	3,94	9,67
IRS2	Insulin receptor substrate 2	11182	N/A	9,45
ITGA5	Integrin, alpha 5 (fibronectin receptor, alpha polypeptide)	10119	0,63	N/A
KAL1	Kalmann syndrome 1 sequence	44617	0,63	9,45
LIMK1	Lim kinase 1	37847	3,94	0
MAP3K5	Mitogen-activated protein kinase kinase kinase 5	36258	0,63	0
MAPK8IP3	Mitogen-activated protein kinase 8 interacting protein 3	14609	3,94	7,14
MMP9	matrix metallopeptidase 9	26338	0	6,93
NDN	Necdin homolog (mouse)	1442	0,63	0
NRAS	Neuroblastoma RAS viral (v-ras) oncogene homolog	3139	3,31	0,21
NRXN1	Neurexin 1	116392	3,31	N/A
PDPK1	3-phosphoinositide dependent protein kinase-1	14469	0	9,45
PRKCA	Protein kinase C, alpha	114871	0	9,45
PTEN	Phosphatase and tensin homolog	4722	1,7	7,36
PTGS2	Prostaglandin-endoperoxide synthase 2	112626	3,94	9,45
PTK2	Protein Tyrosine Kinase 2	116986	0,63	0
PTK2B	Protein tyrosine kinase 2 beta	39829	0	0
PTPN1	Protein tyrosine phosphatase, non-receptor type 1	26382	0	0,21
RB1CC1	RB1-inducible coiled-coil 1	40992	3,31	N/A
RGMA	RGM Domain Family Member B	33277	3,31	N/A
RGMB	RGM Domain Family Member A	13823	3,94	0
RRAS	Related RAS viral (r-ras) oncogene homolog	21730	3,94	7,14
SEMA3B	Semaphorin 3B	29403	0	9,24
SEMA4A	Semaphorin 4A	1354	3,94	9,67
SGK1	Serum/glucocorticoid regulated kinase 1	36274	6,61	6,93
TBX21	T-box 21	17380	0,63	9,67
TP53	Tumor protein p53	19095	3,94	9,45
TPM3	Tropomyosin 3	20450	1,7	9,45
TSC2	Tuberous sclerosis 2	14637	0	0,43
UNC5B	UNC5-homolog b	4599	0	N/A
WASL	Wiskott-Aldirich syndrome-like	38866	6,61	6,93
WT1	Wilm’s tumor 1	8172	0,63	9,45

When Table 3 was further analyzed for genes that could play a role in neuronal differentiation, migration, or axonal guidance, however, a different subset were particularly found to be interesting. Among these, DCLK1 (doublecortin-like kinase) is a protein kinase that is known to be upregulated in response to BDNF signal, and to be involved in neuronal migration and neurogenesis (http://www.ncbi.nlm.nih.gov/gene/9201) [41]; LIMK1 (LIM domain kinase 1) regulates actin cytoskeletal dynamics and was shown to be linked to BDNF-induced neuritogenesis [42]; UNC5B, when bound to netrin-4, is involved in thalamocortical axon branching [43]; and NRXN1 codes for neurexin1 protein that functions in cell adhesion in vertebrate nervous system (http://www.genecards.org/cgi-bin/carddisp.pl?gene=NRXN1).

It should be noted, however, that both manual curation followed by manual *in silico* analysis and automated promoter screening for Pea3/ETV4 binding does not in any way imply that these promoters are genuine targets for Pea3/ETV4 in neurons. Therefore, experimental verification is necessary for both *in silico* approaches.

### Microarray analysis of target genes

To experimentally verify the predictions, as well as to identify novel targets for Pea3/ETV4 in neurons, we have carried out a microarray analysis in SH-SY5Y human neuroblastoma cell lines overexpressing mPea3-VP16 fusion protein. When the data were analyzed, quite surprisingly 68.7% of all affected genes were found to be repressed between 2- and 5-fold in cells overexpressing Pea3-VP16 as compared to pCDNA3-transfected cells, with about 23.3% of all affected genes being repressed over 5-fold. Since VP16 is a highly potent activation domain, such a high ratio of repressed genes could either be explained through an indirect repression via activation of specific miRNA genes (which could not be identified in the arrays employed in this study), or through steric hindrance of a critical transactivator from binding when Pea3-VP16 was bound.

To identify the impact of these changes at cellular level and determine the affected pathways, microarray data were further analyzed in 5 runs of PANOGA. These results were then listed from the most statistically significant pathway to the least: Cell cycle, MAPK signaling pathway and Pathways in cancer, Endocytosis and Neurotrophin signaling pathway appeared in the top five (Table 5). Among the pathways directly related to neural circuit assembly are ECM-receptor interaction and axon guidance pathways, which include genes such as EFNA3, EPHA2, SEMA4C, L1CAM that exhibit high statistical significance in PANOGA analysis (Table 5). Others in these pathways, such as EFNB1, EFNB2, and UNC5A also appear as potential Pea3 targets, albeit with lower significance (p<0.004; data not shown). These genes are of particular interest to this study, since they are reported to be directly involved in neural fold fusion, neural differentiation, or axonal guidance in previous reports [44–48].

**Table 5:** PANOGA analysis of microarray results. Data was run 5 times, and genes with statistical significance were reported for occurrence and name. Pathways of interest are indicated in **bold**.

Pathways	p-value	Occurrence	Affected Genes
Cell cycle	4,18748E-21	5	RB1, PKMYT1, FZR1, CCND1, YWHAQ, E2F1, CDC25A
**MAPK signaling pathway**	**7,39475E-19**	**5**	**MAX, ARRB1, ARRB2, DUSP16, ELK1, RELA, RELB, RPS6KA4, RPS6KA3, MAPK1, RAC3, CACNG2, DUSP4, MAP3K3, JUND, TRAF2, DUSP7, TAOK2, MAP3K11, FGFR1**
Pathways in cancer	1,11864E-17	4	RB1, RET, PIK3R2, RELA, RXRB, CCND1, GNA11, DVL2, E2F1, MAPK1, RAC3, FADD, PLCG1, VHL, RALGDS, APC2, JUP, DAPK3, ARNT, AXIN2, RARA, ARHGEF1, FGFR1
**Endocytosis**	**4,6808E-14**	**5**	**RET, AP2A1, AP2A2, GIT1, SH3GL1, AP2B1, VPS37B, SMAD6, DNM1, EPN1, DNM2, EPN2, RAB11B, SMAD7, CHMP4B**
**Neurotrophin signaling pathway**	**4,2358E-13**	**5**	**MAP3K3, NGFR, SHC1, PIK3R2, RELA, RPS6KA3, ARHGDIA, RAPGEF1, BAX, MAPK1, PLCG1**
Focal adhesion	6,05774E-13	4	LAMA5, SHC1, PIK3R2, ELK1, CCND1, MAPK1, VASP, GRLF1, VAV2, COL6A1, ITGA11, RAPGEF1
Proteoglycans in cancer	3,15804E-12	5	SDC4, PIK3R2, ELK1, CCND1, GPC1, MAPK1, PLCG1, VAV2, RPS6KB2, ARHGEF1, PTPN6, FGFR1
Glycerolipid metabolism	3,82705E-12	1	AGPAT1, AGPAT2, AGPAT4
**SNARE interactions in vesicular transport**	**6,46398E-12**	**3**	**VAMP2**
TNF signaling pathway	1,08568E-11	2	PIK3R2, TRAF2, RELA, RPS6KA4, CREB3L3, MAPK1, JUNB
Chronic myeloid leukemia	1,34911E-11	5	RB1, SHC1, PIK3R2, RELA, CCND1, E2F1, MAPK1
Fc gamma R-mediated phagocytosis	3,0438E-11	5	VASP, SPHK2, PIK3R2, DNM2, VAV2, MAPK1, PIP5K1A, PLCG1, WASF2
Hepatitis B	5,52921E-11	5	RB1, PIK3R2, ELK1, RELA, CCND1, YWHAQ, E2F1, BAX, MAPK1, PTK2B, FADD
Colorectal cancer	1,09676E-10	5	APC2, PIK3R2, AXIN2, CCND1, BAX, CYCS, MAPK1, RAC3, RALGDS
**Apoptosis**	**1,21339E-10**	**4**	**DFFA, PIK3R2, TRAF2, RELA, PRKAR2A, BAX, CYCS, CAPN1, FADD**
GnRH signaling pathway	1,58743E-10	3	MAP3K3, ELK1, PLCB3, GNA11, MAPK1, PTK2B
T cell receptor signaling pathway	1,69056E-10	5	PIK3R2, RELA, VAV2, MAPK1, PTPN6, PLCG1, NFKBIB
Adherens junction	1,88693E-10	5	MAPK1, RAC3, PTPN6, WASF2, FGFR1
Fat digestion and absorption	2,43063E-10	1	AGPAT1, AGPAT2
**Synaptic vesicle cycle**	**2,64159E-10**	**3**	**AP2A1, CPLX2, DNM1, VAMP2**
Epstein-Barr virus infection	3,38556E-10	5	PIK3R2, RELA, RELB, YWHAQ, POLR3H, TAB1
Transcriptional misregulation in cancer	4,4283E-10	3	MAX, MLLT1, RELA, CCND2, RXRG, NGFR, TAF15, JUP, FUS, TFE3, ETV4, RARA, TCF3
B cell receptor signaling pathway	6,64022E-10	5	INPPL1, PIK3R2, RELA, VAV2, CD79A, MAPK1, PTPN6, NFKBIB
**ECM-receptor interaction**	**6,76663E-10**	**2**	**LAMA5, COL6A2, COL6A1, ITGA11**
**Ras signaling pathway**	**7,02702E-10**	**4**	**SHC1, PIK3R2, ELK1, RELA, SYNGAP1, MAPK1, PLCG1, RALGDS, NGFR, EFNA3, RASA3, GNB1, EPHA2**
**ErbB signaling pathway**	**1,05542E-09**	**4**	**SHC1, PIK3R2, ELK1, RPS6KB2, MAPK1, PLCG1**
Basal transcription factors	1,55859E-09	3	TAF6L, TAF15
Bladder cancer	3,01127E-09	5	RB1, CCND1, E2F1
Prostate cancer	4,59634E-09	5	RB1, PIK3R2, RELA, CCND1, E2F1, MAPK1, FGFR1
Non-small cell lung cancer	4,90448E-09	4	RB1, PIK3R2, RXRB, CCND1, E2F1, MAPK1, PLCG1, RXRG
Rap1 signaling pathway	7,80956E-09	5	VASP, NGFR, PIK3R2, ACTG1, VAV2, PLCB3, RAPGEF1, MAPK1, PLCG1, RALGDS, RAPGEF6, EPHA2, FGFR1
Viral carcinogenesis	7,87916E-09	5	RB1, PIK3R2, RELA, MAPK1, SCRIB, BAX
**Regulation of actin cytoskeleton**	**8,30967E-09**	**1**	**CYFIP2, GRLF1, PIK3R2, ACTN4, BAIAP2, FGD1, ACTG1, VAV2, ITGA11, MAPK1, PIP5K1A, ARHGEF1, WASF2, GIT1, FGFR1**
Small cell lung cancer	9,88309E-09	5	RB1, CCND1, E2F1, CYCS, TRAF2, RELA
Chemokine signaling pathway	1,00353E-08	5	SHC1, ARRB1, PIK3R2, ARRB2, RELA, VAV2, PLCB3, ADRBK1, GRK6, MAPK1
Acute myeloid leukemia	1,2986E-08	4	JUP, PIK3R2, RELA, CCND1, RPS6KB2, PIM1, RARA, MAPK1
Pancreatic cancer	2,24346E-08	5	RB1, PIK3R2, RELA, CCND1, E2F1, MAPK1, RAC3
Osteoclast differentiation	2,32427E-08	3	JUND, FHL2, PIK3R2, TRAF2, RELA, RELB, MAPK1, JUNB
Progesterone-mediated oocyte maturation	2,40544E-08	3	PIK3R2, PKMYT1, CDC25A, RPS6KA3, FZR1, MAPK1
Spliceosome	2,46814E-08	2	SF3A2, RBM8A, U2AF1, PRPF19, THOC4, U2AF2
Bacterial invasion of epithelial cells	2,49617E-08	4	SHC1, PIK3R2, DNM1, DNM2, WASF2
Fc epsilon RI signaling pathway	2,98389E-08	3	PIK3R2, VAV2, MAPK1, RAC3, PLCG1
Endometrial cancer	5,11242E-08	4	APC2, CCND1, MAPK1, PIK3R2, AXIN2, ELK1
Proteasome	5,88839E-08	2	
**Wnt signaling pathway**	**9,1423E-08**	**4**	**APC2, FBXW11, AXIN2, CSNK1E, CCND1, DVL2, RAC3**
Shigellosis	9,26388E-08	5	FBXW11, U2AF1, RELA, MAPK1, NFKBIB
**Glioma**	**1,00691E-07**	**4**	**RB1, SHC1, PIK3R2, CCND1, E2F1, MAPK1, PLCG1**
**Notch signaling pathway**	**1,15517E-07**	**3**	**APH1A, NCOR2, DVL2, DTX2**
Thyroid hormone signaling pathway	1,24296E-07	3	GATA4, PIK3R2, ATP1B1, RXRB, CCND1, MAPK1, PLCG1, RXRG
**Hippo signaling pathway**	**2,79196E-07**	**1**	**FBXW11, SCRIB, AXIN2, CSNK1E, SMAD7, CCND1, YWHAQ, DVL2**
Citrate cycle (TCA cycle)	2,98326E-07	1	OGDH
Renal cell carcinoma	3,93043E-07	3	RAPGEF1, ARNT, MAPK1, PIK3R2, VHL
AMPK signaling pathway	5,05726E-07	3	CRTC2, PIK3R2, CAMKK1, STK11, CCND1, FASN, HNF4A, AKT1S1, RPS6KB2
Choline metabolism in cancer	7,23983E-07	4	PIK3R2, DGKZ, DGKQ, MAPK1, PIP5K1A, PLCG1, RALGDS, WASF2
Thyroid cancer	7,26445E-07	3	RET, RXRB, CCND1, MAPK1
Nucleotide excision repair	7,67343E-07	1	LIG1, POLE
Natural killer cell mediated cytotoxicity	1,04223E-06	3	FCER1G, SHC1, PIK3R2, VAV2, SH3BP2, MAPK1, PTK2B, PTPN6, PLCG1
Prolactin signaling pathway	1,11893E-06	3	SHC1, MAPK1, PIK3R2, RELA
Platelet activation	1,18344E-06	4	VASP, FCER1G, PIK3R2, ACTG1, PLCB3, MAPK1, ARHGEF1
Insulin signaling pathway	1,39589E-06	5	SHC1, INPPL1, PIK3R2, ELK1, RAPGEF1, MAPK1
mTOR signaling pathway	1,63553E-06	4	RPS6KA3, STK11, AKT1S1, RPS6KB2, MAPK1, PIK3R2
Vasopressin-regulated water reabsorption	1,80142E-06	1	RAB5C, VAMP2, RAB11B
**Axon guidance**	**2,29678E-06**	**2**	**SEMA4C, L1CAM, EFNA3, MAPK1, RAC3, EPHA2**
Oocyte meiosis	3,11271E-06	2	FBXW11, PKMYT1, YWHAQ, MAPK1
TGF-beta signaling pathway	3,8067E-06	3	MAPK1, SMAD6, SMAD7
Salmonella infection	3,87325E-06	2	PKN3, RELA, KLC3, KLC2, MAPK1
Estrogen signaling pathway	4,48771E-06	2	CALML5, SHC1, PIK3R2, CREB3L3, MAPK1
**Amyotrophic lateral sclerosis (ALS)**	**6,28617E-06**	**2**	**BAX, CYCS, NOS1**
Sphingolipid signaling pathway	6,6267E-06	4	FCER1G, SPHK2, PIK3R2, TRAF2, RELA, PPP2R2B, MAPK1
NF-kappa B signaling pathway	9,00498E-06	2	PIAS4, LRDD, RELA, RELB, LBP, PLCG1
HIF-1 signaling pathway	9,5515E-06	4	ARNT, PIK3R2, RELA, RPS6KB2, MAPK1, PLCG1, VHL
NOD-like receptor signaling pathway	1,02878E-05	1	MAPK1, RELA, NFKBIB
FoxO signaling pathway	1,05545E-05	3	PRMT1, PIK3R2, CSNK1E, STK11
**Glutamatergic synapse**	**1,12921E-05**	**1**	**GRIK5, GRIK3, PLCB3, GRM4, DLG4, ADRBK1, GNB1, MAPK1**
Endocrine and other factor-regulated calcium reabsorption	1,14065E-05	3	AP2A1, AP2B1, ATP1B1, AP2A2, DNM1, DNM2
Melanoma	1,15013E-05	2	RB1, CCND1, E2F1, MAPK1, PIK3R2
Pathogenic Escherichia coli infection	1,21309E-05	2	YWHAQ
**Dopaminergic synapse**	**1,70854E-05**	**1**	**CALML5, ARRB2, CREB3L3**
Epithelial cell signaling in Helicobacter pylori infection	1,88839E-05	1	CSK, GIT1, RELA
Type II diabetes mellitus	2,27294E-05	1	MAPK1, PIK3R2
Leukocyte transendothelial migration	2,50496E-05	3	VASP, GRLF1, PIK3R2, ACTN4, ACTG1, VAV2, PTK2B, PLCG1
VEGF signaling pathway	3,35777E-05	1	SPHK2, MAPK1, PIK3R2
Ubiquitin mediated proteolysis	3,39334E-05	1	PIAS4, FBXW11, PRPF19, FZR1, VHL
Herpes simplex infection	3,52446E-05	1	RELA, PER1, TAF6L, CYCS, FADD, TAB1
Adipocytokine signaling pathway	3,84037E-05	2	RXRB, STK11, TRAF2, RXRG, CAMKK1, RELA, NFKBIB
Chagas disease (American trypanosomiasis)	5,31326E-05	1	PLCB3, PPP2R2B, GNA11, MAPK1, PIK3R2, FADD, RELA
Toxoplasmosis	5,53351E-05	1	RELA, CYCS, MAPK1, NFKBIB
HTLV-I infection	8,18359E-05	1	RB1, CRTC2, PIK3R2, IL2RG, ELK1, RELA, RELB, CCND1, DVL2, E2F1, APC2, EGR1, MAP3K3, BAX, TCF3
**PI3K-Akt signaling pathway**	**8,27352E-05**	**1**	**LAMA5, CRTC2, PIK3R2, IL2RG, RELA, STK11, CCND1, YWHAQ, MAPK1, NGFR, EFNA3, RPS6KB2, EPHA2**
**p53 signaling pathway**	**9,0672E-05**	**2**	**CCND2, CCND1, LRDD, BAI1**

It is important to note that the presence of endocytosis, focal adhesion, SNARE interactions in vesicular transport, synaptic vesicle cycle, and regulation of actin cytoskeleton pathways among the results (Table 5) indicates that Pea3 may also be reinforcing its role in neural circuit assembly through these pathways. Ephrins, for example, were shown to trigger endocytosis in order to mediate repulsion; similarly, Sema3A-mediated growth cone collapse was shown to occur alongside endocytosis (rev. in [49]). Reorganization of the actin cytoskeleton is a sure must in growth cone guidance and/or collapse (rev. in [49]).

Wnt signaling, Notch signaling, and Hippo signaling pathway components, among many others, were also found to be affected in response to exogenous Pea3-VP16 expression (Table 5). Although Wnt signaling was long known for its role in early embryonic development, their role in growth cone and axon guidance have been identified only a decade ago [50, 51]. Notch signaling is involved in the early development of many systems, nervous system being one—it was shown to be important for axonal outgrowth as well as dendritic patterning in various model systems [52–54]. Hippo pathway, which is known to be a common regulator of organ size in development, was recently shown to mediate ephrinB/EphB signaling in peripheral nerve regeneration [55]. Hippo and Wnt pathways have also been shown to cross-talk in various systems [56], and regulate *Drosophila* photoreceptor fate [57].

There were also quite a number of immune system-related pathways affected by Pea3-VP16 overexpression, such as those in Tumor Necrosis Factor (TNF) signaling pathway, Fc gamma R-mediated phagocytosis, and T cell receptor signaling pathway (Table 5). Immune system has been on the stage for quite some time in several processes from neurogenesis to brain tumors and neurodegeneration [58, 59]. TNF, for example, was shown to inhibit neurite outgrowth in the hippocampus [60]. In addition, presence of active T cells were found to be crucial for neural stem cell maintenance in the SVZ [58]. Thus, the fact that a significant number of genes regulated by Pea3 turn out to be immune system-related should be noted.

### Verification of axon guidance pathway and related genes

It should be emphasized that KEGG Pathway database is a collection of manually drawn wiring diagrams for pathways and, while immensely informative, it unfortunately does not cover all genes involved in any particular pathway [61]. We have therefore gone back to the original microarray data in the light of PANOGA analysis, and compared genes identified in the significant pathways with the genes identified in the manually curated data. Some of the *in silico*-identified genes (Tables 3 and 4) were indeed found to be affected in microarray data, including L1CAM, NGFR, PTK2B and EFNB2, to be either up- or down-regulated; others, such as neuron-specific cyclin dependent kinase CDKR51 did not yield a statistically significant result, whereas its close homolog CDK5R2 was found to be repressed by around 2-fold in SH-SY5Y cells, and CDK10 was repressed by around 4-fold (data not shown). Based on these, we have restricted our verification analyses to potential novel targets of Pea3 that could be directly involved in axonal growth, guidance, and neural circuit formation that were common in all three analyses—manual curation, *in silico* automated analysis and microarray (data not shown). Among these are EFNA3, EFNB1, EFNB2, FGFR1, NGFR, PTK2B, SEMA4C, UNC5A, L1CAM, EPHA1, EPHA2, GLUD2 and GRIK3.

**Fig 2:**
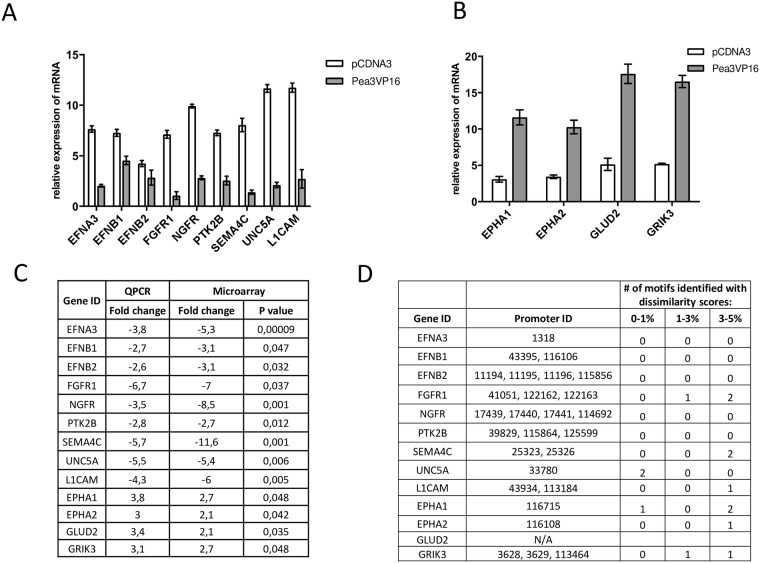
Verification and analysis of a subset of target promoters. (a) q-RT-PCR results for a set of genes that were repressed upon Pea3-VP16 overexpression in SH-SY5Y cells (grey bars) as compared to pCDNA3-transfected cells (white bars); (b) q-RT-PCR results for a set of genes that were activated upon Pea3-VP16 overexpression in SH-SY5Y cells (grey bars) as compared to pCDNA3-transfected cells (white bars); (c) comparison of fold change in q-RT-PCR assay vs microarray results; (d) analysis of promoters for these genes for putative Pea3 binding sites, if available.

Using qRT-PCR assays in SH-SY5Y cells transfected with pCDNA3 or pCMV-mPea3-VP16 expression plasmids, we have first confirmed repression of EFNA3, EFNB1, EFNB2, FGFR1, NGFR, PTK2B, SEMA4C, UNC5A and L1CAM genes when Pea3-VP16 protein was overexpressed (Fig 2a). On the contrary, EPHA1, EPHA2, GLUD2 and GRIK3 were upregulated upon Pea3-VP16 expression (Fig 2b). The fold-changes between qRT-PCR and microarray assays were compared and found to be parallel to each other, ie repressed in both or activated in both, even though the extent of repression or activation may be different due to the resolution and sensitivity of the assay used (Fig 2c). When the promoters for these genes were analyzed for potential Pea3 binding motifs, some (but not all) of the negatively regulated gene promoters did not exhibit a high-affinity binding motif for Pea3, indicating at least some of the repression events may be indirect (Fig 2d; no promoter sequence was available for GLUD2 in the database utilized). Yet, high affinity Pea3 binding sites were predicted in some of the negatively regulated gene promoters, such as FGFR1 and Sema4C, and in some positively regulated gene promoters such as EPHA1 and EPHA2 (Fig 2d). Whether Pea3 can indeed bind to these predicted sites *in vivo* remains to be determined.

### Kallikreins—novel Pea3 targets

**Fig 3:**
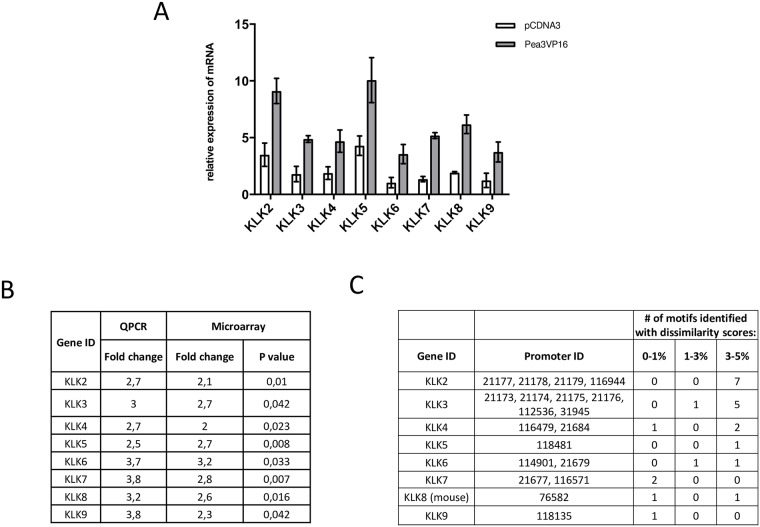
Analysis of kallikreins as novel targets for Pea3. (a) q-RT-PCR results for KLK2-9 that were activated upon Pea3-VP16 overexpression in SH-SY5Y cells (grey bars) as compared to pCDNA3-transfected cells (white bars); (b) comparison of fold change in q-RT-PCR assay vs microarray results; (d) analysis of kallikrein promoters for putative Pea3 binding sites.

A novel set of targets were also identified upon analysis of microarray data, which were not identified through *in silico* studies: kallikreins, serine proteases that cleave peptide bonds in proteins found in many physiological systems. Unlike matrix metalloproteases (MMPs), which are among the known targets of Pea3-dependent transcriptional regulation that degrade mainly extracellular matrix proteins, kallikreins have been implied in degradation of hormones such as somatostatin and pro-insulin (KLK1; [62]), myelin, amyloid peptide, GluR and α-synuclein (KLK6; [62]), L1-CAM (KLK8/neuropsin; [63, 64]), and ephrin-B2 (KLK4; [65]). Using qRT-PCR assays in SH-SY5Y cells transfected with pCDNA3 or pCMV-mPea3-VP16 expression plasmids, we have first confirmed transactivation results seen in microarray for KLK2-9 (Fig 3a). When the fold-activations in qRT-PCR assays were compared to those observed in microarray experiment, they were found to be consistently activated between 2- to 4-fold (Fig 3b). When the promoters of these genes were analyzed, all of them were predicted to contain one or more putative Pea3 binding motifs that exhibit 0–5% dissimilarity (Fig 3c). KLK2 and KLK3, which are largely studied with respect to prostate cancer (Lisle et al, 2015) showed large number of relatively low-affinity Pea3 motifs, whereas KLK6 and KLK8, shown to cleave α-synuclein and L1-CAM, respectively, had higher-affinity binding motifs (Fig 3c). Whether Pea3 directly binds to and regulates these promoters in neurons remain to be studied, however it should be noted that KLK8, for example, was shown to induce neurite growth and fasciculation of hippocampal neurons as well as formation and maturation of synaptic boutons in Schaffer collateral pathways, and to regulate Schaffer collateral long term potentiation (LTP) in hippocampus [63–68], suggesting kallikreins, particularly KLK6 and KLK8, as novel transcriptional targets of Pea3.

### Binding of Pea3 on promoters

One rather interesting and surprising result of microarray experiments that could not be foreseen through *in silico* analyses was the large set of genes that were repressed upon Pea3-VP16 overexpression in SH-SY5Y cells (data not shown). Some of the repression events were then confirmed through qRT-PCR (Fig 2). One explanation could be the switch of Pea3 ETS protein from an activator to a repressor through SUMOylation [69–71]. However, since VP16 is a highly potent transactivator, the repression observed was thought to be through an indirect mechanism, where Pea3-VP16 activates a global repressor or a miRNA gene. This is a likely mechanism, because the promoters of some of the repressed genes analyzed exhibited no high-affinity binding sites for Pea3 (Fig 2d).

**Fig 4:**
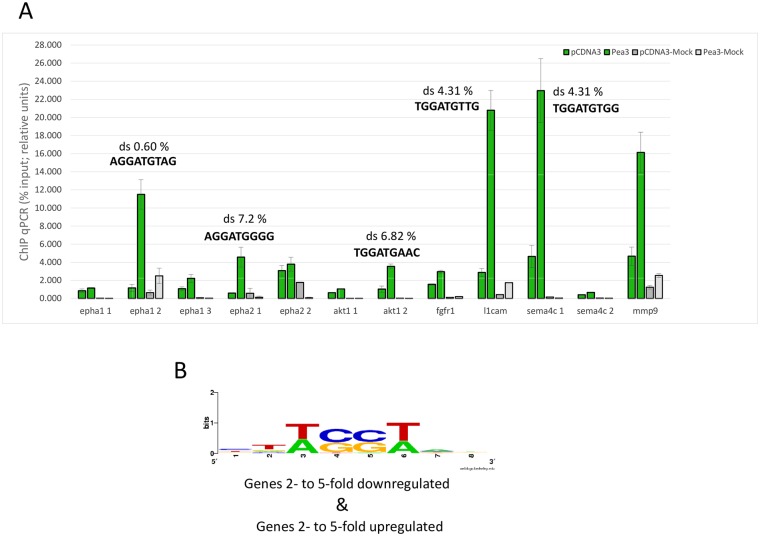
Chromatin immunoprecipitation (ChIP) and Pea3-VP16 binding. (a) ChIP PCR in untransfected vs Pea3-VP16 overexpressing SH-SY5Y cells, immunoprecipitated with either Flag antibody (Flag IP) or IgG (IgG IP). Dissimilarity score (ds) of selected *ets* motifs are indicated, and explained further in text; (b) weblogo analysis for genes that were either 2- to 5-fold downregulated or2- to 5-fold upregulated upon Pea3-VP16 expression in SH-SY5Y cells.

To confirm whether Pea3 can directly or indirectly bind to the identified subset of promoters, we have conducted chromatin immunoprecipitation (ChIP) assays on some of the *ets* motifs identified through *in silico* promoter analyses (Fig 2d). Indeed, Pea3-VP16 was found to bind both *epha1* and *ehpa2* promoters, albeit with different intensities on different *ets* motifs (Fig 4a). *Epha1* promoter was found to have one *ets* motif with dissimilarity score (ds) smaller than 1% (ds 0.60%), and two *ets* motifs with dissimilarity scores between 3 and 5% (Fig 2d). Pea3-VP16 showed higher binding to the former motif (epha1 2), and lower binding to the latter two (epha1 1 and epha1 3), as expected from *in silico* prediction (Fig 4a). *Epha2* promoter had slightly lower binding of Pea3-VP16 to the epha2 1 motif, which in fact contains two tandem *ets* motifs with relatively high dissimilarities (ds 7.42%, shown in Fig 4a, and ds 10.54%, not shown); epha 2 2 motif has a higher ds score than epha2 1, reflected in ChIP assay; Fig 4a).

Similarly, *l1cam* and *sema4c* promoters were also confirmed to bind Pea3-VP16, in spite of the fact that *ets* motifs of both promoters show high dissimilarity rates (Figs 2d and 4a; ds 4.31%). *Akt* promoter contained two *ets* motifs, one of which showed a stronger binding to Pea3-VP16 in ChIP assays (Fig 4a; ds 6.82%), and the stronger *ets* motif of *fgfr1* promoter also indicated Pea3-VP16 binding (ds not shown) Other target promoters from different KEGG pathways were also found to give higher qPCR results in ChIP assays, such as *cxcr4*, *rhoA* and *elk-1* promoters (data not shown). *Mmp9* promoter was used as a positive control for Pea3 binding (ds 0%, Fig 4a [72]).

We have then analyzed promoter regions for up- or down-regulated genes for putative Pea3 binding motifs, and analyzed these sites using WebLogo tool for common patterns. When promoters of genes that were up- or down-regulated 2- to 5-fold were separately analyzed, the classical GGA core motif [2,73] was observed in both groups (TCCT/AGGA; summarized in Fig 4b). These motifs were also confirmed in the limited ChIP assays (Fig 4a). However, when promoters of genes downregulated 5-fold or more were grouped and analyzed separately, the putative Pea3 binding motifs predicted, if any, were quite far from the consensus 5’-AGGAAG-3’ binding site ([2]; ACGT/TGCA; data not shown), indicating an indirect repression mechanism by Pea3 (see Conclusion).

## Conclusion

ETS transcription factors were shown to be regulated in a temporally regulated manner at later stages of nervous system development, in particular for normal sensory neuron differentiation and during branching [74]. Pea3 family of proteins are expressed from E9.5 till birth, and in some cases after birth, starting with brain regions followed by expression in lung, thymus, cartilage and mammary tissue of mouse [75]. Pea3 and Er81 appear to be particularly important at later stages of neural development, whereas Erm seems to be involved in early differentiation of neural crest stem cells [76].

Glial-derived neurotrophic factor (GDNF) as well as Met signaling were shown to regulate the expression of Pea3 proteins in prospective motor neurons, and in a mutually exclusive manner in subpopulations of motor neuron pools [77–79]. Fibroblast growth factors (FGFs) were also shown to regulate Pea3 subfamily members during development at various brain regions and retina [15, 80]. In the retina, FGF was shown to regulate Pea3 expression in a MAPK-dependent manner, resulting in expression of neurofilament-M, which was also confirmed to be a Pea3 target by our laboratory [6, 7, 15]. In the chick, FGF3-dependent upregulation of Pea3 was shown to be important for Krox20-dependent hindbrain segmentation [81]. It should be noted that no significant change in Krox20/EGR2 was observed in our microarray analysis, whereas a repression of around 7-fold was seen on EGR1 levels (data not shown).

In spite of many reports on the role and importance of Pea3 subfamily members in nervous system development, only cadherin-8, Ephrin receptor 4 (Ephr4), semaphorin-3E and neurofilaments were previously shown to be targets of Pea3 [7, 16, 18]. In *C*. *elegans*, ETS protein Ast-1 (axon steering defect-1) was shown to be responsible for dopaminergic neuron differentiation, with loss of *ast-1* causing the failure of all dopaminergic neurons to terminally differentiate [17]. In this system, Ast-1 was shown to regulate major dopaminergic pathway genes through a dopamine (DA) motif, although a counterpart function for Pea3 subfamily member Er81/ETV1 is not yet confirmed for mammalian dopaminergic system [17].

In this study, we have developed an automated tool for identification of potential novel target promoters for regulation by given transcription factors, which we have used to identify novel Pea3 target genes; the analysis was further supported by microarray studies. Our results indicate that such *in silico* tools can indeed identify genuine binding sites with significant accuracy, yet fail to indicate whether such a binding would result in activation or repression. In the microarray analysis presented in this study, we have identified novel targets of Pea3 transcription factor that are both down- and up-regulated. Our chromatin immunoprecipitation studies analyzed direct binding of Pea3 to a small subset of promoters, and parallel q-RT-PCR assays confirmed some of the repressions observed in microarray experiments (Figs 2 and 4). Earlier studies indicate that, while mostly known as transactivators, ETS proteins can act as repressors depending on post-translational modification status, such as SUMOylation [71]. Therefore, such post-translational modifications on Pea3 fusion partner of Pea3-VP16 protein may also affect transcriptional regulation of target promoters. Additionally, binding of Pea3-VP16 to these promoters may be sterically hindering a crucial transactivator from binding, thereby causing a repression of a subset of genes outside a rather narrow developmental window, ensuring timely expression of such critical genes. Another explanation could be post-translational modifications of Pea3, since similar modifications such as SUMOylation have been known to convert some ETS family members to repressors [69–71].

In addition to components of Wnt, Notch and Hippo pathways, genes within Endocytosis, Synaptic vesicle cycling and Immune pathways were also found to be potential targets of Pea3 in microarray analysis (Table 5). Extensive analysis is required to further illuminate the mechanism and relevance of these potential targets for neural circuit formation.

**Fig 5:**
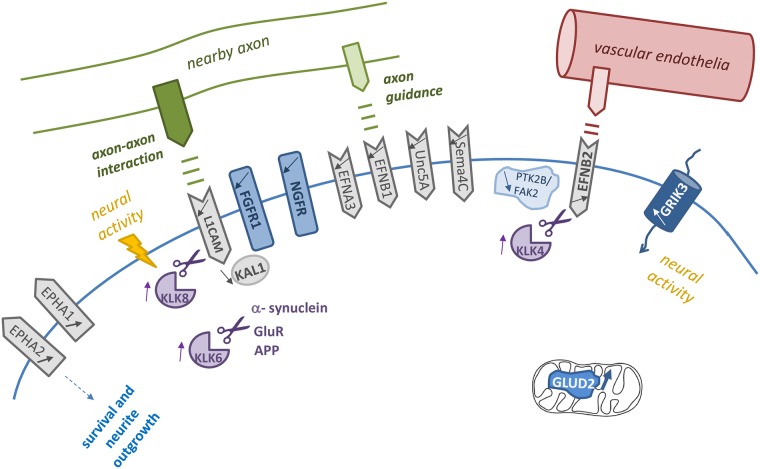
A schematic summary of genes that were identified to be novel targets for Pea3 analyzed in this study, along with their interactions and functions; L1CAM, EFNA3, EFNB1, Unc5A and SEMA4c are involved in axon-axon interactions and axonal guidance; KLK8 activated by neural activity was shown to degrade L1CAM, and KLK4 was reported to cut EFNB2, while KLK6 was shown to cleave α-synuclein, GluR and APP. EFNB2 on vascular endothelial cells helps maintain stem cell identity. FGFR1 and its partner KAL1, involved in post-mitotic development of neuronal cells, are both downregulated. EPHA1 and EPHA2, both upregulated, are involved in survival and neurite outgrowth. GLUD2 and GRIK3, both correlated with neuronal activity, are upregulated in Pea3-VP16-expressing cells.

In line with a relatively late-stage function of Pea3 in nervous system development, it appears that genes related to axonal guidance or axon-axon interaction are down-regulated, directly or indirectly, whereas genes related to survival, neurite outgrowth and maturation of synaptic boutons, as well as neural activity were upregulated (Fig 5). While Sema4C is downregulated (Fig 2a and 2c), plexin A1, a co-receptor for semaphorins, is also downregulated (around 5-fold; data not shown). Among the genes identified in microarray experiments, EFNA3, for example, was shown to be expressed in primitive streak in early mouse embryos [46], and EFNB2 plays a role in early cortical development [48], both of which are down-regulated upon Pea3-VP16 expression in microarray and qRT-PCR studies (Fig 2a and 2c), whereas EPHA1 and EPHA2, involved in neurite outgrowth and post-natal neuromuscular junction formation [82] are up-regulated (Fig 2b and 2c). These data support earlier reports that Pea3 family members were functional at late stages of neuronal circuit formation [83]. Having said that, the story of ephrins and ephrin receptors in neurons appears to be more complicated—for example, EphB2, the receptor for ephrin B, is important for synaptic signaling and LTP formation [82] and EPHA2 was shown to be important in mammalian neural precursor cell (NPC) differentiation and neurogenesis [45], yet EFNB1 and EphA2 together were found to play a role in neurite outgrowth. EFNB2 on the membranes of vascular endothelial cells, on the other hand, blocks cell cycle entry in order to maintain stem cell identity [84]. Hence, more in-depth analysis of how different Pea3 family members dynamically regulate different ephrins and ephrin receptors in a spatiotemporal manner is required.

Nonetheless, it is intriguing that kallikrein KLK8 is upregulated upon Pea3 expression, while at the same time its substrate L1CAM is downregulated (Figs 2, 3 and 5). Similarly, as KLK4 was upregulated, its substrate EFNB2 was downregulated by Pea3 (Figs 2, 3 and 5). No such parallels were found between KLK6, which was upregulated (Figs 3 and 5), and its substrates APP (no significant change; data not shown) or a-synuclein (no significant change; data not shown). One of the predicted KLK6 substrates is glutamate receptor GluR [62], yet excitatory ionotropic glutamate receptor GRIK3 (otherwise known as GluR7) was upregulated nearly 3-fold (Fig 2b and 2c), and metabotropic glutamate receptor GRM4 was upregulated around 2.5-fold (data not shown). Why both the enzyme and its substrates are up-regulated at the same time is yet unclear, however the fact that many other excitatory ion channels such as nicotinic cholinergic receptor CHRNA2 is upregulated by around 2.5-fold, while inhibitory chloride channel CLCN7 was downregulated by around 12-fold (data not shown) indicates an active role for Pea3 in neuronal activity upon terminal differentiation. Also upregulated are genes involved in synaptic vesicles, such as synaptotagmin (2.8-fold), those involved in neurotransmitter release, such as DOC2A (2.5-fold), and myelination, such as myelin oligodendrocyte glycoprotein (MOG, 2.5-fold) (data not shown). Some of the genes identified in this study can also explain the involvement of Pea3 family members in many forms of cancer. KLK2, 3, and 5 have all been implicated in prostate cancer, whereas KLK9 is implicated in both prostate and breast cancers, where Pea3 has been associated with [4, 13, 41]. Therefore we believe that this combinatorial approach to identifying novel targets of Pea3 not only will help us understand its role in nervous system, but also in progression of many types of cancer.

We would also like to emphasize that experimental as well as *in silico* assays and different algorithms such as that presented in this study could be used complementary to genome-wide microarray analyses so as to narrow down target identification and eliminate possible false negatives or wrong identifications.
